# Evaluation of cardiac hypertrophy in the setting of sudden cardiac death

**DOI:** 10.1080/20961790.2019.1633761

**Published:** 2019-08-19

**Authors:** Kristopher S. Cunningham, Danna A. Spears, Melanie Care

**Affiliations:** a Department of Laboratory Medicine and Pathobiology, Ontario Forensic Pathology Service, University of Toronto, Toronto, Canada;; b University Health Network, Division of Cardiology – Electrophysiology, University of Toronto, Toronto, Canada;; c Fred A. Litwin Family Centre in Genetic Medicine and Inherited Arrhythmia Clinic, University Health Network & Mount Sinai Hospital, University of Toronto, Toronto, Canada

**Keywords:** Forensic sciences, forensic pathology, ventricular hypertrophy, arrhythmia, sudden cardiac death, genetics

## Abstract

Ventricular hypertrophy is a common pathological finding at autopsy that can act as a substrate for arrhythmogenesis. Pathologists grapple with the significance of ventricular hypertrophy when assessing the sudden and unexpected deaths of young people and what it could mean for surviving family members. The pathological spectrum of left ventricular hypertrophy (LVH) is reviewed herein. This article is oriented to the practicing autopsy pathologist to help make sense of various patterns of increased heart muscle, particularly those that are not clearly cardiomyopathic, yet present in the setting of sudden cardiac death. The article also reviews factors influencing arrhythmogenesis as well as genetic mutations most commonly associated with ventricular hypertrophy, especially those associated with hypertrophic cardiomyopathy (HCM).

## General principles

Myocardial hypertrophy is a common finding at autopsy. It represents a pathological substrate that is associated with arrhythmogenesis and for the forensic pathologist may help explain why on individual may die suddenly and unexpectedly. Myocardial hypertrophy may not only underlay some sudden, natural deaths, but also deaths that occur by accidental means, such as following sudden loss of consciousness while operating a motorized vehicle or a large piece of machinery in the workplace or under circumstances where there is struggle and restraint by police at the time of apprehension. Forensic pathologists frequently face the circumstance where an individual is witnessed to die suddenly and unexpectedly or is found deceased, despite appearing entirely well a short time earlier. In the absence of a clear alternative cause for death following evaluation of all information available, including a complete autopsy with additional ancillary studies where appropriate, such as toxicology, biochemistry and microbiology, it is reasonable to consider that death (or the circumstances leading to death) may be best explained as the result of an acute arrhythmic event caused by some underlying cardiac substrate. This is where the careful consideration of any myocardial hypertrophy identified is warranted.

Significant left ventricular hypertrophy (LVH) increases the risk of sudden cardiac death 6- to 8-fold in men and 3-fold in women. In patients with definite electrocardiographic (ECG) evidence of LVH there is a 59% overall mortality at 12 years. LVH is an independent risk factor for coronary artery disease and increases the risk of congestive heart failure 10-fold by 16 years [1]. The incidence of myocardial hypertrophy will likely increase at the time of autopsy as the age of the population increases and the proportion of individuals with chronic comorbidities such as obesity [2], hypertension [1] and chronic kidney disease [3] escalates in western society. Finally, LVH elevates the risk of myocardial infarction as well as cardiovascular morbidity and mortality in patients with hypertension [1]. Thus, myocardial hypertrophy is a stable, chronic pathological substrate that is well recognized to increase the risk of ventricular arrhythmias, cardiac failure and sudden cardiac death. Nevertheless, the spectrum of how myocardial hypertrophy can present at autopsy and the significance of such hypertrophy for the death investigation may not be fully appreciated by all pathologists. When most physicians undergo their training, cardiac hypertrophy is often presented as being concentric in nature and caused by increased afterload impedance from conditions such as hypertension or aortic stenosis. Yet concentric LVH represents only one pattern of cardiac hypertrophy that may be observed at autopsy. As a result, pathologists may not “see” other forms of hypertrophy when they encounter it and understand what such hypertrophy says about the pathophysiological processes that were at play in the months to years prior to death and at what stage in its natural history the myocardial disease is being evaluated. Moreover, being able to interpret the macroscopic and microscopic findings within the myocardium enables the pathologist to proffer an opinion on the severity of the disease present, whether the pathology identified is adequately explained by the clinical history available (i.e. whether it correlates to the nature of the disease under consideration or to the timescale expected by the history available) or when identified in a younger person, whether there may be an underlying genetic contribution to the disease. This article seeks to address many of these issues and provide guidance to the pathologist when having to interpret myocardial hypertrophy. In addition, we provide an account of the nature of the arrhythmogenesis that may result from the myocardial disease and the genetic issues to contemplate when a primary cardiomyopathic process may be at hand.

## Introduction

Inferring the cause of death from a chronic, stable pathological substrate such as myocardial hypertrophy may be causally challenging at the level of the individual case. It is recognized that sudden death from conditions associated with significant LVH is not rare. In addition, in many autopsies where myocardial hypertrophy is identified, the severity of the myocardial disease is more than sufficient to increase the risk of arrhythmogenesis, the history often coheres with a sudden cardiac death and the remaining postmortem examination (including ancillary studies) fails to identify an alternative cause for death. Arguably the severity of the myocardial disease identified was the same as it was the week or month prior to a given person’s death. Thus, functionally there was likely a new circumstance, such as physical exertion, physiological stress, emotional distress or a drug effect, as well as the establishment of a pro-arrhythmic pathway within the myocardium that may help explain why a given person died when they did. The challenge arises for the pathologist when one is dealing with an apparent sudden cardiac death and the only significant finding is a borderline degree of myocardial hypertrophy; in other words, was there sufficient pathological substrate to support arrhythmogenesis?

Depending on the circumstances of the death and the age of the decedent, other causes of sudden cardiac death, such as a primary arrhythmia syndrome, may need to be considered, even though the heart is not “structurally normal” as is often the dogma that is taught when considering channelopathies as a potential cause for death. When trying to establish the most reasonable cause for death, much is dependent on the age of the individual, the medical history available and an understanding the scene and circumstances of the death. Furthermore, the pathologist must be cautious when inferring the functional severity of any myocardial disease that is identified at autopsy in the period prior to death. For example, while the macroscopic and microscopic features of the myocardium may speak to the severity of the pathological process that was present at the time of death, there is an imperfect overlap between the quality of cardiac performance (or degree of dysfunction) and the severity of the myocardial disease identified at autopsy. We certainly recognize instances where an individual presents with very severe myocardial disease pathologically and the decedent was clinically asymptomatic prior to death and may not have even known they had severe heart disease. The same may be said in some instances for the opposite circumstance, where there was a history of severe congestive heart failure and the myocardial pathology identified at autopsy was very modest. Furthermore, the nature of heart failure that may have been present, whether with preserved ejection fraction (HFpEF) from a typically hypertrophied and/or stiff myocardium or reduced ejection fraction (HFrEF) [4] cannot always be clearly inferred from the postmortem findings alone. Often there may have been elements of both given the enlarged, hypertrophied and remodeled hearts that are frequently seen at autopsy. Undoubtedly, correlation between antemortem clinical history and postmortem findings provides the best chance of elucidating the true nature of the cardiac pathology present and its functional consequences when trying to understand an individual’s sudden cardiac death.

The pathologist must also recognize common physiological causes of hypertrophy. These are generally mild in nature and can occur following chronic endurance exercise [1], within the right ventricle during late foetal and early neonatal life [5] and within maternal hearts during the mid to late intrapartum and postpartum periods [6]. The myocardial thickness may also *mildly* increase in late middle aged and elderly individuals (complete with a mild increase in interstitial fibrous tissue deposition) as part of normal aging. It is interesting to note that the type of endurance exercise may impact the nature of the cardiac hypertrophy that is identified [7]. In adults, physiological hypertrophy typically correlates with an increase in cardiac mass by 10%–20% and is *not* associated with increased interstitial fibrous tissue deposition, chronic degenerative changes or cell loss [8]. In adults, when considering left ventricular thickness at autopsy, a thickness of >1.5 cm is considered abnormal (more later); yet physiological hypertrophy should typically not cause the left ventricular myocardium to exceed 2 cm in thickness. Furthermore, increased wall thickness associated with a diminishment in the size of the chamber should prompt one to consider pathological hypertrophy instead of physiological hypertrophy in most instances. Echocardiographically, in athletes wall thicknesses of >12 mm in males or >10 mm in females are considered abnormal in Caucasoid populations. Athletes of African or Afro-Caribbean ancestry are considered abnormal with >14 mm in males and >11 mm in females [9]. All hypertrophy represents an adaptive response to changing hemodynamic circumstances in an attempt to increase contractility and decrease wall stress. It is now recognized that both physiological and pathological hypertrophy are each underpinned by different molecular mechanisms [8]. For the pathologist, differentiating between physiological and pathological hypertrophy at autopsy is accomplished in part by understanding the clinical history and circumstances as well as evaluating the nature of the pathological findings present.

This is not necessarily an elementary exercise. For instance, the sudden and unexpected death of a woman 2 weeks after delivering a child should not be caused by physiological hypertrophy. Misinterpreting physiological hypertrophy as hypertensive heart disease for instance (particularly where there was no history of hypertension either before or during pregnancy) misses the likely cause of death, which may yet be cardiac (e.g. such as a primary arrhythmia syndrome) or non-cardiac in nature. Nevertheless, following the death of a young person, *unexplained* hypertrophy or hypertrophy that is more severe than expected for the circumstances (i.e. hypertrophy out of context) should raise suspicion of a possible primary cardiomyopathic process, which entails the pathologist bank a source of DNA and recommend that first-degree family members consider referral to a heritable heart disease clinic. The guideline produced by the National Association of Medical Examiners, amongst others are useful references when considering collection of tissue or blood for DNA banking and genetic testing when necessary [10]. In some jurisdictions genetic testing is initiated by the death investigation system. This is best accomplished when done in collaboration with regional cardiologists and genetic counsellors who are responsible for follow-up care of surviving family members. Careful consideration of the needs of these clinics is paramount when arranging for testing. Moreover, selection of what genetic tests are performed requires a balance between providing adequate genetic breadth to cover the spectrum of disease under consideration and limitation of the genetic screens to those genes that may be effectively interpreted and actionable for families. In current practice this is largely accomplished with commercially available, comprehensive next generation sequencing panels that combine those genes most commonly associated with primary arrhythmia syndromes and cardiomyopathies. However, when the pathological diagnosis of the myocardial disease is very specific, such as for hypertrophic cardiomyopathy (HCM) or arrhythmogenic cardiomyopathy, one could choose to significantly limit the testing panel to those most relevant genes. Regular consultation with clinical colleagues in this domain not only provides for better outcomes for families, but facilitates the development of an effective molecular autopsy program to address sudden cardiac deaths in the era of precision medicine.

In regard to myocardial hypertrophy, unfortunately confusion sometimes arises with nomenclature. For instance, cardiomegaly and cardiac hypertrophy are not interchangeable concepts. Cardiomegaly is simply an enlarged heart. One could have a dilated cardiomyopathy with markedly enlarged chambers and a thin myocardial wall; the heart would exhibit cardiomegaly, but not myocardial hypertrophy. It is acknowledged that most hearts that exhibit cardiomegaly also exhibit myocardial hypertrophy and often some degree of ventricular remodeling, but it should be recognized that cardiomegaly is not an aetiologically specific diagnosis and it is simply a global descriptor of the heart. The more serious challenge with nomenclature arises between “hypertrophy” and “hypertrophic cardiomyopathy”. For some clinicians and pathologists who do not understand the difference, these concepts become interchanged, which leads to the impression that a heart with mere myocardial hypertrophy from whatever cause gets labelled as being due to HCM. HCM is an aetiologically specific diagnosis characterized by marked cardiomyocyte hypertrophy, geographic regions of myofibre disarray, myocardial scar and in most cases is likely associated with an underlying genetic anomaly. When a death is attributed to HCM, it entails a recommendation by the pathologist or coroner that first-degree family members be assessed in a heritable heart disease clinic because of the potential for heritability. This would not be appropriate if the decedent died of hypertensive heart disease, valvular disease or some other acquired cause for the cardiac hypertrophy and would lead to undue anxiety for the family and cost to the family or health care system to investigate surviving family members.

There are three primary patterns of myocardial hypertrophy that are observed at autopsy: concentric, eccentric and asymmetric. Other non-specific patterns of hypertrophy are also observed by the pathologist that are often characterized by regional wall changes associated with conditions such as atherosclerotic heart disease where significant segments of myocardium undergo a “compensatory” hypertrophy in response to separate regions of myocardium that are poorly or no longer contractile due to scar tissue. The patchy myocardial hypertrophy and accompanying global change in chamber dimensions that inevitably follows represent myocardial remodeling following regional myocardial injury. Nevertheless, most cases of myocardial hypertrophy observed at autopsy that globally involve the left ventricular myocardium conform to the concentric, eccentric and asymmetric phenotypes.

Concentric LVH, represented by an increase in the ratio of the wall thickness to the chamber dimension (Table 1), may be observed in both acquired conditions and primary cardiomyopathic processes. Pathologists readily recognize this pattern because of their training, although it is too frequently assumed to represent evidence of hypertensive heart disease when observed. Hypertensive heart disease should only be considered when there is a clinical history of hypertension and/or good evidence of hypertensive damage to other end organs, such as the white matter microvasculature in the brain and the kidneys and no competing cause for this pattern of hypertrophy. Any process that chronically increases afterload (pressure overload within the myocardial chamber) can cause a concentric pattern of hypertrophy, such as systemic arterial hypertension, aortic (subvalvular, valvular or supravalvular) stenosis and coarctation. Even intermittent pressure overload can lead to cardiac hypertrophy [11], which may explain the concentric patterns seen with cocaine or other stimulant abuse and regular static exercise such as weight lifting. Anabolic steroid usage [12–14] as well as the concentric variant of HCM is also associated with a concentric pattern of LVH. It is thought that there is a predominant increase in the number of sarcomeres arranged in parallel versus in series within cardiomyocytes [1]. Furthermore, there is often increased interstitial and perivascular fibrous tissue deposition, particularly within the subendocardial third of the left ventricular myocardium. While most of the increased mass of the heart is related to the increased size of cardiomyocytes, increased fibrous tissue and other supportive cellular and non-cellular elements contribute. Finally, increased wall thickness relative to the chamber may also be observed in earlier stages of some infiltrative diseases, such as cardiac amyloidosis.

**Table 1.** Typical patterns of left ventricular myocardial hypertrophy observed under different conditions.^a^

Patterns	Conditions
Concentric	Hypertension Aortic stenosis (subvalvular/valvular/supravalvular) Coarctation Concentric variant of hypertrophic cardiomyopathy Static exercise (e.g. weight lifting) Anabolic steroid/performance enhancing drug usage Collagen vascular disease (e.g. systemic sclerosis) Infiltrative disease (e.g. amyloid) Storage disease (e.g. Fabry disease)
Eccentric	Aortic insufficiency (consider in setting of ascending aortic aneurysms) Mitral insufficiency Anemia Obesity Chronic kidney disease with haemodialysis Dynamic exercise (e.g. swimming) Congenital heart disease with volume overload Dilated cardiomyopathy

^a^Many conditions could be identified in either category depending on when observed in their natural history.

Eccentric hypertrophy is a pattern of myocardial change where there is actually a decrease in the ratio of the wall thickness relative to the chamber size. This pattern of ventricular hypertrophy is not rare (Table 1). The left ventricular chamber undergoes enlargement (one form of remodeling), although there will also be a significant increase in amount of myocardium that is present to enclose that larger chamber. This may result in a wall thickness that is only modestly increased over what is normal, yet there is an increase in the overall quantity of myocardium and thus, the mass of the heart is increased. Often because the left ventricular chamber undergoes significant enlargement with surrounding myocardium, the right ventricular myocardium also undergoes remodeling. Sarcomeres are thought to be added predominantly in series versus in parallel, causing elongation of the cardiomyocytes [1]. Like the situation in concentrically hypertrophied hearts, the natural history is development of an increased amount of interstitial and perivascular fibrous tissue deposition throughout the myocardium. Yet, while there may be a somewhat greater predilection for fibrous tissue within the subendocardial third of the left ventricular myocardium, it is often not as severe as what can be seen in hearts that undergo significant concentric hypertrophy. An eccentric pattern of hypertrophy is often underappreciated at the time of autopsy by some pathologists, because the changes to the wall thickness and remodeled chamber may not be as visually striking as what can be seen in concentric hypertrophy or with asymmetric septal hypertrophy. As such, unless the pathologist is aware of this pattern of hypertrophy the heart may be interpreted as “normal” given the normal anatomical relationships that are preserved and lack of a localized myocardial pathology present, even though the heart is significantly heavier than what is expected. Chronic volume overload is the hemodynamic condition that promotes development of an eccentric form of LVH.

Asymmetric septal hypertrophy is commonly associated with the classical variant of HCM. Here the interventricular septum is markedly thickened relative to the free wall; although often the free wall can also exhibit significant hypertrophy. There may be significant variability in the regional wall thickness with HCM. Less common variants of HCM may be seen with regional wall thickenings involving the mid-septal wall, the apex, the anterior-lateral free wall and posterior-basal septum/free wall. Portions of the left ventricular wall may be of normal thickness and only show subtle histological changes. Thus, regions of myocardium exhibiting myofibre disarray are quite variable with a distribution that may be patchy or that may diffusely involve the myocardium, including the right ventricular myocardium. There is also a variable degree of myocardial scar, both interstitial and replacement-type scar, observed within the myocardium of both ventricular chambers, although there does seem to be a predilection for the basal muscular interventricular septum or regions of greatest hypertrophy. Anecdotally, we see a number of cases of HCM at autopsy, either incidentally or in association with a sudden cardiac death, with a concentric (symmetric) pattern of hypertrophy. In children, LVH resembling HCM may be observed as part of other systemic conditions, such as Noonan Syndrome, mitochondrial myopathies and metabolic disorders, which are unrelated to sarcomeric protein mutations [15]. Finally, more advanced forms of HCM may appear as a heavily scarred, dilated cardiomyopathic phenotype, which is sometimes referred to as a “burnt out HCM”. In some instances, this phenotypic appearance may not initially be recognized as representing HCM echocardiographically and only be properly diagnosed at the time of autopsy when the marked cardiomyocyte hypertrophied myofibre disarray is noted histologically.

A few words of caution for the pathologist are warranted. It should be recognized that virtually all forms of myocardial disease undergo structural changes as part of their natural history. This includes hypertrophy and remodeling (typically an enlargement) of the respective chamber involved and often secondarily involves other cardiac chambers due to the volume and pressure changes that may result (such as left atrial or right ventricular enlargement). Moreover, a given disease process may be associated with a concentric pattern of hypertrophy in the earlier course of its natural history, whereas more advanced forms of disease may evolve to a more eccentric pattern. Ultimately, the end stage of most if not all forms of chronic myocardial disease is a dilated cardiomyopathic phenotype. Thus, the stage in the natural history you happen to examine the heart may dictate what pattern of hypertrophy you observe. For pathologists, having access to echocardiography reports as part of the clinical history provides additional points in time when the heart can be evaluated structurally and may assist in understanding how the heart has changed over time. As such, it is important to not be dogmatic about what differential diagnostic considerations are possible for any particular pattern of hypertrophy observed, but rather recognize that the concentric and eccentric patterns are simple guides to help elucidate potential aetiologies of disease. Both patterns may be present at different times for a given disease process. As always, when interpreting the cause for a particular form of hypertrophy, it is important to contextualize this with other autopsy findings and clinical record.

A second issue that occasionally arises at the time of autopsy is that a heart may have been examined by echocardiography in the recent past (typically transthoracic) and interpreted as “normal”, only to find that it exhibits significant enlargement and increased mass (often with an eccentric pattern of hypertrophy) at autopsy. Echocardiographic measurement of LVH and myocardial mass has a specificity and sensitivity of 80% [1]. Although ECG testing and echocardiographic testing can reveal the presence of LVH, the agreement between the two modalities may be poor, suggesting that each may convey distinct risk information regarding LVH [16]. As such, for the death investigator reviewing medical records, evaluating either test in isolation to screen for the presence of LVH is not recommended. Magnetic resonance imaging is considered the clinical gold standard to determine LVH and mass in life and is much preferred for comparison with autopsy findings when reports are available. Thus, at the early stages of a death investigation, it is likely unwise to use prior echocardiography reports to help evaluate whether a heart is likely to be normal or not at autopsy. Furthermore, in the age of postmortem computed tomography (CT) scanning prior to autopsy, which can be used to help triage cases and determine whether dissection may be indicated, the appearance of the heart by CT scanning after death does not typically provide sufficient information to infer the presence or absence of significant myocardial disease, including myocardial hypertrophy.

A more general issue that needs comment is that the closer we look at the myocardium in the era of increased diagnostic sensitivity to genetic cardiovascular disease, the more natural variability in the gross and histological appearance of the heart muscle that is appreciated by pathologists, and the more frequently we must evaluate pathological findings of unknown significance. Thus, when macroscopic or microscopic changes are identified outside of the range of expected normal variability, the significance of such changes can be a challenge to interpret at the time of autopsy. One may evaluate a heart displaying none or only modest macroscopic evidence of hypertrophy, yet microscopically there may be significant cardiomyocyte hypertrophy with atypical, sometimes bizarre cardiomyocyte nuclei exhibiting an excess of pleomorphism and hyperchromasia for what would be expected for the age of the decedent, the size of the heart and the clinical history available. There may also be idiopathic fibrous tissue deposition out of context for the history available. In some instances this may be associated with multiple foci of myofibre disarray or markedly atypical myocyte architectural arrangements. In the setting of a sudden cardiac death where no other historical, anatomical or toxicological findings explain the death, these changes indicate the presence of diseased myocardium and would very reasonably be concluded to increase the risk of ventricular arrhythmia and sudden cardiac death. However, what pathophysiological process precisely represents is less clear.

In the Papadakis study looking at a series of apparent sudden cardiac deaths for which no clear pathological substrate was identified to explain the death, there was a significant number of cases that showed some degree of myocardial hypertrophy/idiopathic fibrous tissue deposition that was not easily explained and did not fit into the classical diagnostic entities we recognize pathologically [17]. In addition, subsequent mutational analysis revealed abnormities in genes classically associated with primary arrhythmia syndromes. What is the significance of these mutational changes in the setting of uncertain pathological findings? The authors suggest that the structural changes identified may represent incidental findings in people with primary arrhythmia syndromes (innocent-bystander), which may better explain the nature of the sudden death or may indicate that structural findings may represent an independent pathological process that acts as a trigger for an underlying primary arrhythmia syndrome [17]. Alternatively, these structural changes may represent a poorly characterized structural variation that can be linked to some primary arrhythmia syndrome or subtle cardiomyopathy. Much is yet unknown from the cardiovascular pathology perspective with respect to some deaths in young people. In other words, cardiologists and cardiovascular pathologists likely do not have a complete appreciation of the full range of myocardial pathologies that are possible in the deaths of young people. In a forensic pathology practice that sees an enrichment of cases of sudden cardiac deaths in young people, uncommon forms of myocardial disease that do not conform to typical diagnostic entities are occasionally identified and it is not clear how to interpret their significance with respect to the cause of death and with respect to biological family members. Proper characterization of these findings is particularly important when trying to ascertain if someone may (or may not) have an underlying genetic condition. For the pathologist dealing with such challenging cases, the best approach may be to remain descriptive and not presuppose a specific diagnosis in the absence of additional information. Furthermore, in some circumstances the best individuals to ascertain the significance of these uncertain pathological findings may be a cardiologist and geneticist/genetic counsellor when first degree family members are later evaluated in clinic; this is particularly the case when genetic anomalies are identified in concert with the pathological findings. There is certainly a need for more basic research to better characterize such myocardial pathology and it will be of particular interest to evaluate these changes in the context of any genetic anomalies that may also be present.

## How might one think about cardiac hypertrophy at autopsy?

While there are different macroscopic patterns of myocardial hypertrophy that are observed at autopsy, microscopically, with the exception of a few diagnostic entities the changes observed are generally non-specific with respect to aetiology. For instance, the histological appearance of the left ventricular myocardium from an 800 g heart in a 26-year-old with idiopathic dilated cardiomyopathy may be identical to an 800 g heart in a 60-year-old with chronic aortic insufficiency. Establishing the most likely underlying cause for the hypertrophy comes from integrating all pathological findings, circumstances and clinical history. The severity and distribution of disease may differ from one case to another, but it is often not possible to infer the underlying aetiology from the histology of the left ventricular myocardium alone. Integration of the information gained from the structure of the heart at the time of autopsy, the clinical history, the age of the decedent and any cardiological investigations performed in the past may be necessary to establish a reasonable diagnosis. Nevertheless, there are exceptions to this premise, for instance: infiltrative disease such as sarcoid, amyloid and hemochromatosis or storage disease processes such as Pompe disease or Fabry disease will be diagnostically characteristic (or strongly suggestive) at autopsy, which can be further investigated with supplementary ancillary studies. In addition, marked cardiomyocyte hypertrophy associated with geographic regions myofibre disarray, sometimes along with architectural disarray of muscle bundles, as well as potentially aberrant microvascular changes are supportive of an HCM.

As indicated above, when the ventricular myocardium undergoes hypertrophy, histologically the cardiomyocytes become enlarged and acquire changes to their nuclei. When assessing nuclear changes it is useful to remember that an internal size control exists with myofibroblast or endothelial nuclei located within the interstitium. Normal cardiomyocyte nuclei are typically round to ovoid, non-hyperchromatic with smooth edges and perhaps 3–5 times the size of an endothelial cell nucleus. With cardiac hypertrophy nuclei enlarge significantly, become hyperchromatic and pleomorphic in their appearance and size, they take on variable shapes that include angulations (classically described as a “box-car”-like morphology although other variations exist). With conditions such as HCM the nuclei can become markedly enlarged, typically much larger than what is seen in most forms of LVH and sometimes quite atypical in their morphology.

Associated with cardiomyocyte hypertrophy are a number of changes to the interstitium. When the myocardium remodels, it is not only the cardiomyocytes that changes, but the interstitium remodels as does the microvasculature that supplies the heart muscle. As disease progresses there is an increase in interstitial and perivascular fibrous tissue deposition. Often this fibrous tissue is diffuse and non-specific in its appearance. Alternatively, the fibrous tissue may have a predilection for a specific region of the myocardium, such as within a specific vascular territory or within the subepicardium, mid-myocardium or subendocardium. These patterns of fibrous tissue deposition can be very diagnostically useful when assessing hypertrophied myocardium and formulating a view to the differential diagnosis. Epicardial predominant fibrous tissue deposition within the left ventricular myocardium, particularly when associated with fibrofatty tissue infiltration, may be indicative of arrhythmogenic cardiomyopathy. Subendocardial predominant fibrous tissue can be seen under circumstances where there has been a prior ischaemic insult to the myocardium. This could be due to concomitant coronary artery disease, but marked ventricular wall thickening alone can also be associated with cardiomyocyte loss and replacement with fibrous tissue within the subendocardium. It is recognized that as the ventricular myocardium undergoes hypertrophy there is a relative rarefaction of the microvasculature, indicating that the density of the microvasculature does not keep up with the increase in muscle mass. Specifically, coronary microvasculature rarefaction associated with a reduced microvascular density and the resultant reduction in coronary flow reserve renders the myocardium susceptible to ischaemia, particularly under conditions of physiological stress [18, 19]. Thus, myocardial regions classically thought of as vascular watershed zones in the heart, such as the subendocardium, the tips of the papillary muscles, the posterior-basal wall and the basal, muscular interventricular septum are susceptible to cardiomyocyte dropout and fibrous tissue deposition from chronic, low grade ischaemic injury. Moreover, pressure overload leading to increased myocardial mass leads to increased oxygen demand, which requires further metabolic and mechanical adaptations. In the setting of aortic stenosis for example, significant myocardial hypertrophy is seen to decouple the balance between blood flow and myocardial demand and adversely affects blood flow within the epicardial coronary arteries and microvasculature, which also contributes to chronic myocardial ischaemia [20].

With myocardial hypertrophy, not only is there remodeling of cardiomyocyte architecture, but cardiomyocyte metabolism, induction of gene programs to increase expression of foetal gene expression, increased mitochondrial dysfunction, altered sarcomeric structure, altered ion channel expression and impaired calcium handling are also identified [8]. The remodeling myocardial interstitium results in altered interactions between the interstitium and cardiomyocytes, increased fibrous tissue deposition and impaired angiogenesis [8, 21, 22]. Finally, factors promoting myocardial hypertrophy often also promote chronic neurohormonal activation of the renin-angiotensin-aldosterone system as well as the sympathetic nervous system. Such activation can promote vasoconstriction, apoptosis as well as fibrous tissue deposition [8]. Thus, when considering the underlying substrates promoting arrhythmogenesis in the setting of ventricular hypertrophy, there are clearly macroscopic factors, microscopic factors and molecular/metabolic factors that must be evaluated.

When assessing the myocardium histologically following the autopsy, the presence of increased interstitial or even replacement-type fibrous tissue deposition should be seen as a cumulative change that progresses as disease severity increases. Thus, the presence of significant fibrous tissue deposition with cardiomyocyte dropout and occasional cardiomyocytes exhibiting chronic degenerative changes should be seen as advanced disease. The presence of advanced fibrous tissue deposition (moderate to marked in severity) and myocardial degenerative changes may be useful pathological markers of advanced disease when assessing whether someone may be at increased risk of sudden and unexpected death. With advanced disease it may be difficult to pathologically separate advanced ventricular hypertrophy with remodeling from frank cardiomyopathy, particularly if the functionality of the heart (e.g. ejection fraction of the left ventricle) is unknown. Both may be associated with a reduction in the ejection fraction, although it is likely better to think of the myocardial phenotype as cardiomyopathic when there is significant reduction with cardiac performance. With respect to sudden cardiac death, although there is some evidence to suggest that the lower the ejection fraction (particularly if <35%), the greater the risk of sudden and unexpected death, this is an imperfect measure as a significant proportion of sudden cardiac deaths occur in patients with ejection fractions >40%, with significant differences noted between ischaemic and non-ischaemic pathologies [23].

## What are the challenges of interpreting cardiac hypertrophy at autopsy?

When does one actually have sufficient myocardial hypertrophy at autopsy to potentially cause death? There is not a straightforward answer to this question and opinions vary somewhat amongst pathologists depending on the circumstances. If we start from the position that we have what appears to be a sudden and unexpected death with no alternative cause for death identified and the only significant finding is the presence of myocardial hypertrophy, then there are a few factors that may assist in this deciding if there is sufficient hypertrophy to cause death. First, significantly increased cardiac mass is likely the best measure of severity of the degree of LVH present. One has to factor in any increase in mass contributed by a hypertrophied right ventricle as well (if present), but significant increases in mass will predominantly be left ventricular in origin. It is important to consistently remove any excess soft tissue and vascular tissues from the heart when establishing the mass, such as trimming off the superior and inferior caval veins, trimming the excess aorta and pulmonary trunk distal to the valves as well as removing any pericardium and attached mediastinal soft tissues. Although there will clearly be variability in the amount of epicardial adipose tissue present from person to person, it is not recommended that this tissue be removed systematically as each pathologist will differ in their completeness and this procedure is not generally performed when assembling data for reference tables (see below). Moreover, although there are recognized, older techniques requiring dissection of each of the left and right ventricular myocardium from the remainder of the heart and weighing these individually, this is time consuming, subject to the same variability in individual practice and is seldom of great utility. It is well recognized that the heart mass exhibits plasticity and adjusts in size to the individual in question, the sex of the individual and in response to any physiological or pathophysiological influence present. Thus, when evaluating the mass of the heart, these factors must be considered. The role of racial ancestry and what is considered normal in hearts is not entirely clear either. Classically it is thought that individuals of African or Afro-Caribbean ancestry may have larger hearts with modestly thicker ventricular walls compared to other groups. However, when considered across populations, some studies fail to identify statistically different values for heart mass at the time of autopsy [24]. It is likely best to consider the sex, the size and relative muscularity of each individual in question when considering what a normal heart is for that individual. Certainly when considering the heart masses of college age competitive athletes in the US who die suddenly and unexpectedly, hearts larger than 500 g are seen that do not otherwise exhibit a specific myocardial pathology [25].

There are no perfect reference tables when considering heart masses in men and women [26–29]. Each study is biased to some degree for different reasons based on the types of cases considered (e.g. variability in medical histories of the patients included), the distribution of the height and weight of the populations that are autopsied or the age distribution of the individuals autopsied in the sample group. One of the more popular reference tables were those compiled by Kitzman et al. [29] from the Mayo Clinic looking at “normal” ranges for both men and women based on height or body weight. However, the challenge with this study is that the reference ranges for heart mass are very broad, likely overestimating what is considered normal as a consequence of including persons with pre-existing morbidities and across a broad age range. While it is acknowledged that the heart mass can increase very modestly as an age-related change, perhaps not to the degree reflected in some of the tables available from the past. More recent studies with better designs were created by Molina and DiMaio [30, 31] for both men and women. These studies considered populations who were 18–35 years of age and died from traumatic causes with no pathological or clinical history of cardiac disease, history of illicit drug usage, history of prolonged medical treatment, history of prolonged time between injury and death, history of cardiac injury or history of systemic disease. For women, the authors proposed a normal range of 148–296 g for those with a body mass index in the normal range. For those with body mass indexes >30 the heart weight average was 305 g with a range of 192–422 g. For men, the authors proposed a normal range of 233–383 g for those with a body mass index in the normal range. For those with body mass indexes >30, the heart weight average was 377 g with a range of 273–575 g. Thus, the range of heart weights considered normal for each sex is significantly narrowed compared to the Kitzman study and there is a clear increase in the distribution of heart masses, particularly at the upper end that is associated with increased body weight, where the heart would not necessarily be expected to be normal because of obesity associated changes (particularly for the morbidly obese). The DiMaio tables are certainly of value when assessing what is normal for heart masses in younger adults, those individuals that are of greatest difficult to ascertain if there could be an underlying genetic anomaly causing hypertrophy.

Most forensic pathologists would consider heart weights >500 g (in the non-muscular, non-competitive athlete) as being both significantly abnormal and likely to place the decedent at increased risk of sudden death. Another measure to consider when assessing if a heart is abnormal is total heart weight greater than 30% over the predicted norm [15]. Difficulty arises when individuals have a heart mass <500 g yet greater than the normal ranges for each sex. Presumably the heart is transitioning from normal to abnormal during the earlier stages of some disease process, yet the degree of hypertrophy is not clearly, potentially lethal and is somewhat circumstance dependent in its interpretation. If the decedent was a male with a height of 177.80 cm, a history of hypertension and a heart mass of 425 g, then the heart was likely exhibiting evidence of early hypertensive heart disease, yet not likely enough to account for death. If the decedent was a male with a height of 195.58 cm and athletic build, then a heart mass of 450 g may not necessarily represent evidence of pathological hypertrophy; however the same could not be said about a woman with a height of 157.48 cm. Thus, there clearly will be some degree of subjectivity in assessing borderline cases and any opinion should be correlated with the entire circumstantial and clinical history as well as other autopsy findings.

Finally, the presence of significant end-organ damage (significant cardiomyocyte hypertrophy, degenerative changes and fibrous tissue deposition) detected histologically in the myocardium of borderline cases may assist in the determination of if the hypertrophy identified was potentially significant as these are histological markers for more advanced disease. Paediatric heart weights for infants and younger children appear to show somewhat greater predictability when compared against body weight, although the same problems likely arise with older children and adolescents with increased body mass index and other comorbidities that are seen with adults. Certainly for infants and younger children within normal body weight ranges and appropriate growth for age, the tables available in Scholz et al. [32] are helpful.

When the myocardial hypertrophy was clearly sufficient to increase the risk of ventricular arrhythmia and sudden death, then determining if it did in fact cause death depends on an evaluation of all circumstantial factors, clinical history and autopsy findings. The question distils down to: did the person *die of* a cardiac disease process or *die with* a cardiac disease process? This too is not always an easy determination. It is dependent on the quality and completeness of the history available to the pathologist and what other competing causes for death are under consideration.

Sometimes the circumstances of the death are quite helpful in determining if a death was likely arrhythmic or not, for instance, if the death was witnessed to be sudden and unexpected in an individual who just appeared well prior to death, versus someone who is clearly sick and unwell in the hours or days prior to death. In other instances a potentially new intervening factor, such as a potentially lethal quantity of an intoxicant or an infection or a metabolic abnormality best explains death over a stable, chronic pathological substrate that does not exhibit evidence of a recent pathological change. Such evaluations are not always perfect and each individual pathologist must balance the evidence as best fits the death investigation.

It is normal for there to be some variability in the thickness of the left and right ventricular myocardium at autopsy. The muscular interventricular septum is typically a little bit thicker than the left ventricular free wall, with a ratio of septum to free wall of 1.1. With left ventricular thickening caused by hypertensive heart disease, Fabry disease or amyloidosis, occasionally we see this ratio increases modestly. Caution is warranted not to over interpret the asymmetry of left ventricular thickening that can be observed under such conditions as representing HCM, as this has been noted to occur anecdotally by echocardiography, which was ultimately not confirmed at the time of autopsy. The right ventricular myocardium also exhibits some variability in the wall thickness between the anterior-lateral wall along the acute margin, the ventral wall, the posterior-basal wall and the right ventricular outflow tract. The latter two are typically somewhat thicker than the former. At autopsy, the right ventricular myocardial thickness is measured at the basal to mid ventricular level and should not exceed 0.3–0.5 cm and normally tapers as it approaches the apex. Measurements should always be of the compact myocardial layer and should not include the trabeculae carnae and care should be taken to avoid epicardial fat. The left ventricular myocardium is also measured at the basal to mid ventricular level (typically about 2 cm from the mitral annulus in adults) and is normally 1.2–1.5 cm in thickness [15]. Measurement should only include the compact myocardial layer and not include trabeculae carnae and epicardial fat. Moreover, special care not to include trabeculae carnae on either side of the muscular interventricular septum should be made when taking this measurement. Abnormal measurements for the right and left ventricular myocardium are conventionally set at >0.5 cm and >1.5 cm, respectively at the time of autopsy. These measurements are somewhat thicker than what is identified echocardiographically, when luminal pressures compress the myocardium and a normal left ventricular thickness is <1.2 cm [33]. Nevertheless, because of the normal variability of thickness of different regions of the left ventricular myocardium and perhaps the state of contraction of the heart at the time of death, the most reliable assessment to gauge the degree of hypertrophy is to interpret the overall mass of the heart as outlined above.

A few comments should be made about sampling the heart for histology. First, it is wise to avoid the junctions of the free wall with the muscular interventricular septum if possible (unless there is a specific pathological finding one wants to evaluate at those locations) as in these regions there is normally a degree of myofibre disarray and sometimes fibrous tissue, particularly in the posterior septum. There can also be very focal myofibre disarray present in normal hearts where papillary muscles insert into the free wall as well as for large trabeculae carnae within the right ventricular myocardium. For those who are not aware of these naturally occurring sites of disarray, one may over interpret the histological appearance of the myocardium as evidence of HCM. Features to keep in mind that are helpful when encountering this issue are: (i) Cardiomyocytes in HCM are markedly enlarged, far more than what is typical for hypertensive heart disease or aortic stenosis; (ii) One wants to see geographic regions of myofibre disarray, typically across multiple sections of the heart to call it HCM. While the myofibre disarray associated with HCM need not involve all regions of the myocardium, it does not present as highly localized, minute disease of the myocardium. Finally, occasional small foci of chronic inflammation can be seen in the setting of HCM and should not be over interpreted as evidence of a lymphocytic myocarditis.

Occasionally the severity of the LVH may suggest one type of cardiac pathology over another. For instance, a 40-year-old man with a 5-year history of hypertension dies suddenly and unexpectedly. At autopsy the heart has a mass of 850 g. While this man may certainly develop hypertensive heart disease given this clinical history, caution should be warranted given the relatively young age of the decedent, the limited time that he had clinical hypertension and the marked degree of hypertrophy present in an 850 g heart. Although there are certainly exceptions that are recognized, most hearts with masses >800–850 g are due to conditions such as longstanding atherosclerotic heart disease, valvular disease with elements of volume overload, obesity associated heart disease with longstanding morbid obesity, adult congenital heart disease or cardiomyopathies. In a 40-year-old man with only a 5-year history of hypertension, in the absence of other findings it may be reasonable to consider that this pathological process may better represent evidence of a primary cardiomyopathy instead of hypertensive heart disease.

In some instances, pathologists may not recognize that the cardiac hypertrophy present (±remodeling) may help to explain death in the setting other cardiac pathologies. Examples include: (i) congenitally bicuspid aortic valve with severe stenosis and LVH, the risk of sudden death is tied to the degree of LVH and not merely the presence of a bicuspid valvular architecture; (ii) sudden death in the setting of mitral valve prolapse where often there is significant hypertrophy of the anterior-lateral and posterior-medial papillary muscles as well as hypertrophy and increased fibrous tissue deposition within the free wall (particularly the posterior-basal wall) of the left ventricular myocardium; (iii) congenital heart disease with significant hypertrophy and remodeling due to chronic pressure and volume overload physiology. While most children do quite well following surgery for congenital heart disease and survive to adulthood, the presence of significant hypertrophy and remodeling that develops as a result of the congenital heart disease or its treatment can increase the risk of sudden arrhythmic death, particularly in the adult population [34, 35]; and finally (iv) delayed postoperative sudden death following aortic valve replacement. Here the sudden and unexpected death may be attributable to the ventricular hypertrophy that remains even though the aortic valve was functioning well. Aortic valve replacement leads to decompression of the left ventricular chamber and ultimately some degree of appropriate remodeling of the chamber with reduced hypertrophy, but much of the fibrous tissue that was present typically remains and the risk of sudden death does not decrease to normal [36, 37].

The following scenario illustrates a pitfall that pathologists can fall to at the time of autopsy: a 38-year-old healthy man died suddenly and unexpectedly from what appeared to be a sudden cardiac death. The postmortem examination revealed a heart with a mass of 850 g with an eccentric pattern of hypertrophy. A fusiform ascending aortic aneurysm was also noted within the ascending aorta, although this did not appear to be ruptured, nor was there evidence of an acute aortic dissection. Because of the markedly enlarged heart with severe hypertrophy, the cause of death was provided as hypertensive heart disease.

Unfortunately such cases are not rare in a busy autopsy service. While the pathological substrate likely responsible for triggering the sudden death was interpreted correctly, namely the marked myocardial hypertrophy, the underlying cause for this hypertrophy was not. In this particular instance, identifying the correct underlying cause for the myocardial hypertrophy is very important as it may impact surviving family members. Ascending aortic aneurysms often involve the aortic root. If the aortic root was significantly ectatic as a result, then there may have been chronic aortic insufficiency. In this instance, given the ascending aortic aneurysm (most often fusiform in structure), the size of the heart, the age of the decedent and eccentric pattern of hypertrophy present, chronic aortic insufficiency was the most likely principal cause for the LVH identified (particularly in someone with no reported history of hypertension). It must be stressed that mild aortic insufficiency or mild pathological changes to the aortic root are unlikely to lead to enough volume overload to cause marked LVH; thus, the degree of hypertrophy must correlate with the severity of the ascending aortic and aortic root disease. The reason recognizing this pattern of disease is so important is because this man was an otherwise healthy person who had an unexplained ascending aortic aneurysm. When examined histologically, the wall of these aneurysms often exhibit moderate to marked medial degenerative changes, which in a younger individual can indicate the presence of an underlying aortopathy. Such connective tissue disorders often have an underlying genetic anomaly, which can be heritable [38]. Thus, while the immediate substrate for the arrhythmia that led to the sudden death was the LVH, the underlying cause for the death was likely aortic disease from a heritable aortopathy. Surviving family members need referral to a medical genetics clinic and DNA needs to be banked for any future genetic testing. These cases end up being a challenge for some pathologists to detect because unlike aortic stenosis, the structural changes to the aortic root and valve that lead to aortic insufficiency are more difficult to identify at autopsy if the pathologist is not familiar with them. Furthermore, unlike an echocardiogram there is no easy way for the pathologist to functionally assess the aortic valve. Ectasia of the root, redundancy and thinning of the cusps and often thickening of the free margins of the cusp relative to the cusp bodies can be helpful pathological findings when assessing for chronic aortic insufficiency.

## Special cases

### Idiopathic (left) ventricular hypertrophy

Idiopathic (left) ventricular hypertrophy is a pathological category of heart disease in need of better characterization. It is unclear if these enlarged and hypertrophied hearts, typically found in young people, represent a single clinical entity, a phenotypic variant of HCM (without the pathognomonic histological feature of myofibre disarray, which would upend current histological diagnostic criteria for HCM) or a common phenotype for a number of pathophysiological processes not yet characterized. This pathological entity is identified in a number of studies in association with the sudden and unexpected deaths of young people, and has been studied in athletes [25, 39, 40], although it is not a condition limited to athletic individuals. Macroscopically the hearts typically exhibit a moderate degree of concentric LVH and occasionally the hypertrophy is noted to also involve the right ventricular myocardium. Some authors have used the standard of heart weight ≥50% of the expected mean based on sex, age and body size for weight using the Mayo nomograms [29] or if heart weight is ≤50% of the expected mean, then features suggestive of cardiomyopathy, including LV wall thickness >1.6 cm, increased interstitial fibrous tissue deposition and significant cardiomyocyte hypertrophy [39] are also accepted. Histologically the hearts show significant cardiomyocyte hypertrophy along with all of the typical nuclear changes, often with increased fibrous tissue deposition that is not otherwise explained (i.e. hypertrophy and fibrous tissue out of context). Importantly though, despite extensive sampling there is no evidence of myofibre disarray, or evidence of any other pathological processes such as infiltrative disease, systemic sclerosis, mitochondrial myopathy and storage disease. Performance enhancing drugs should also be considered in athletes. While physiological hypertrophy is certainly recognized in competitive athletes, as indicated above, idiopathic ventricular hypertrophy is associated in the sudden deaths of high school and collegiate level competitive athletes as well as non-athletes, which should preclude this entity representing an extreme form of physiological hypertrophy. Under current classification, it is not appropriate to categorize this entity as HCM in the absence of geographic regions of myofibre disarray.

#### Cardiomyopathy, not otherwise specified (NOS)

In some instances, the sudden death of an individual may be associated with a myocardium that exhibits evidence of significant cardiomyocyte hypertrophy histologically, along with degenerative changes, interstitial fibrous tissue deposition and even occasionally small foci of myofibre disarray/architectural disarray of muscle bundles, yet the heart may be in the normal size range or only modestly enlarged. With only occasional, small foci of myofibre disarray it would not be appropriate to diagnose this entity as an HCM. Importantly, the heart does not exhibit the typical phenotypic patterns of hypertrophic, dilated, restrictive or arrhythmogenic cardiomyopathy, yet based on the histological appearance is clearly cardiomyopathic in nature. This reinforces the need for cardiac histology on all cases of unexpected death and that one cannot use the structural phenotype of the heart to screen out the presence of a cardiomyopathic process.

An interesting study by Tseng et al. [41] examined the autopsy characterization of sudden cardiac death in San Francisco County and found a decreasing prevalence of coronary disease and an increasing prevalence of non-ischaemic causes for sudden cardiac death, including hearts with unexplained hypertrophy, idiopathic forms of cardiomyopathy as well as HCM. It is reasonable to infer that some of these cases may have an underlying genetic anomaly, particularly when they present in young people who die suddenly and unexpectedly. In some instances the degree of non-ischaemic scar that presents within the myocardium is the predominant histological finding in young people [42, 43] who have normal heart masses or hearts with only modest hypertrophy and limited remodeling of the ventricular chambers. The non-specific myocardial scar can be found variably throughout the thickness of the myocardium, although there may be a predilection for the posterior wall and subepicardial region of the left ventricular myocardium, extending into the mid-myocardial layers. The right ventricular myocardium may also be involved. In some cases, occasional small foci of fibrofatty tissue replacement are identified. It is not unreasonable to consider many of these hearts as likely representing a variant of biventricular, arrhythmogenic cardiomyopathy; however, careful consideration of other primary cardiomyopathic processes or acquired causes for myocardial damage, such as myocarditis, drug induced myocardial damage or collagen vascular disease associated cardiomyopathic changes is required. A careful evaluation of other findings at autopsy and the clinical history can assist in narrowing down the differential diagnostic considerations.

Finally, when one encounters a heart that exhibits cardiomyopathic findings that are atypical or unexplained at the time of autopsy, a systemic myopathy also needs to be considered. Skeletal muscle should be sampled for histology.

### Obesity associated heart disease

Obesity associated heart disease is a common finding at autopsy; however, it may be under recognized by pathologists as a potential explanation for sudden cardiac death. In obese individuals, particularly those who are morbidly obese, the heart can undergo a number of structural changes due to a state of chronically high cardiac output, which includes LVH (typically thought to be an eccentric pattern from volume overload, although a concentric pattern can be seen) and ventricular remodeling. There is a positive correlation between the severity of obesity and measures of left ventricular mass. Hypertension, which is present in 50%–60% of obese individuals, is also a likely contributing comorbid factor for some [44, 45]. Neurohormonal and metabolic changes associated with obesity may also contribute to the changes observed in the myocardium [2]. Right heart involvement may flow from left heart disease, particularly if there was diastolic dysfunction present, however the concomitant presence of obstructive sleep apnoea may also contribute to right heart disease. Obesity is recognized as a strong predictor of sudden cardiac death and has been associated with delayed ventricular repolarization. Furthermore, QTc and QTc dispersion were significantly prolonged in overweight and obese individuals, which are subsequently shortened in those individuals who lose weight [46]. The presence of other comorbidities may clearly impact the structure of the myocardium and perhaps influence ventricular repolarization such as hypertension, metabolic syndrome and diabetes mellitus [47, 48]. Perhaps the most common error pathologists may make in regard to obesity associated heart disease is to assume that all obese individuals with an enlarged and hypertrophied heart had hypertension and thus, diagnose the sudden death as being due to hypertensive heart disease. It is important to consult the clinical (and perhaps the medication) history to ascertain if the decedent was likely also hypertensive, at which time one could proffer a diagnosis of obesity and hypertensive heart disease as a cause for death. Finally, because of the prevalence of obesity in western society, it is important to carefully consider the clinical and family history in obese individuals to be sure another non-ischaemic form of heart disease may not be responsible for death. For example, the sudden death of a young, obese person with a significant history of palpitations or syncope or perhaps a strong family history of premature cardiac death or heart failure should prompt a re-evaluation of the differential diagnostic considerations prior to ascribing death as due to obesity itself. This may entail the collection of a source of DNA to investigate the presence of a cardiomyopathy or a primary arrhythmia syndrome.

## Arrhythmogenesis in HCM

In HCM, patchy, multifocal replacement fibrosis can be detected in regions of hypertrophy [49]. Myocardial fibrosis is an important contributor to arrhythmogenesis in HCM, and has been shown to be an independent predictor of arrhythmia outcomes [50–53]. Myocardial fibrosis results in gap junction remodeling [54], down-regulation of the main cardiac connexin Cx43 [55], and dysregulation of ion channels [56, 57], resulting in conduction slowing, unidirectional block, and heterogeneity of refractoriness. Thus, the fibrotic myocardium in HCM creates vulnerability to reentry, and the ability to sustain ventricular tachycardia, after a triggered event, typically a ventricular premature beat (VPB). The greatest potential for reentry exists in the setting of patchy fibrosis, where regions of pronounced conduction block mingle with strands of normal myocytes with intact conduction [58, 59]. In addition, myofibroblasts may also have direct pro-arrhythmic effects on myocytes. There are *in vitro* observations, which have stimulated active investigation of *in vivo* arrhythmogenesis. Myofibroblast-cardiomyocyte coupling seen in cell culture results in myocyte depolarization, after-depolarization, and ectopic arrhythmia generation [60, 61] a phenomenon that may depend on myofibroblast transforming growth factor β-1 (TGF-β1) [62, 63], TGF-β1 may also promote arrhythmogenesis in cardiac myocytes through alteration of RNA transcription of sodium and potassium channel proteins in a rat model. In this model, TGF-β1 resulted in prolongation of action potential duration and early after-depolarizations [64].

There are several mechanisms underlying myocardial ischaemia in HCM, including reduced coronary flow reserve, blunted myocardial blood flow and microvascular ischaemia [65–67]. Coronary artery remodeling is seen in HCM, resulting in reduced coronary artery volume to myocardial mass ratio, and myocardial ischaemia [68]. Ischaemia can lead to arrhythmogenesis by inducing intracellular K^+^ loss, extracellular acidosis and membrane depolarization [69], and by later generating heterogeneity in myocardial repolarization and subsequent phase 2 reentry [70, 71]. The pathogenesis of apical aneurysm formation in HCM is likely multifactorial [72], but microvascular ischaemia and subsequent scar formation contribute substantially to arrhythmia risk [73]. In this setting, ventricular tachycardia (VT) is typically mapped to the heterogeneous border zone of the apical aneurysm scar [74].

Calcium (Ca^2+^) hypersensitivity at the molecular level is well documented in HCM, and pathogenic variants in the genes encoding myofilament proteins have been shown to increase their Ca^2+^ sensitivity, but the specific mechanisms associated with these protein alterations are not yet clear [75]. Proposed mechanisms by which inherited abnormalities in myofilament proteins can lead to changes in intracellular calcium handling include altered myosin ATPase activity [76–79], interference with protein kinase-A (PKA) mediated phosphorylation [80, 81], Ca^2+^/calmodulin-dependent protein kinase II (CaMKII)-dependent phosphorylation of phospholamban (PLN) [82], and alteration of Ca^2+^/Mg^2+^ protein binding sites [83]. Enhanced Ca^2+^ myofilament binding results in increased end-diastolic free Ca^2+^, potentiating the release of calcium from the sarcoplasmic reticulum after brief pauses, resulting in after-depolarizations and triggered activity [84]. The role of after-depolarizations in arrhythmogenesis, particularly in the setting of a substrate capable of sustaining reentrant arrhythmia, is well known [85]. Animal models have demonstrated a link between Ca^2+^ hypersensitivity and ventricular arrhythmias [86–88]. In one mouse model of troponin T mutation the risk of developing ventricular tachycardia was directly proportional to the degree of Ca^2+^ sensitization caused by the troponin T mutation, and this could be reversed by myofilament desensitization. Ca^2+^ sensitization in this model resulted in variability of action potential duration, shorted ERPs, and increased heterogeneity in conduction velocity at elevated heart rates [88]. Another mouse model of troponin T mutation showed evidence of destabilization of the ryanodine receptor (RYR2), a protein with a critical role in controlling release of Ca^2+^ from the sarcoplasmic reticulum, resulting in triggered arrhythmia events [87]. Vulnerability to arrhythmia can depend on the location of the gene variant, [75] as demonstrated in limited studies of human-induced pluripotent stem cell (hiPSC)-derived cardiomyocytes [89].

## The genetics of HCM

HCM is recognized to be the most common hereditary heart disease and is the most common cause of sudden death in the young [90]. Numerous genes are known to cause HCM, with pathogenic mutations identified in 35%–60% of affected individuals [90–92]. Genetic testing for HCM is readily available through commercial and academic genetics labs and the identification of a causative gene mutation can provide both valuable diagnostic information as well as allow for accurate risk stratification and tailored clinical surveillance for family members [90, 93, 94]. The majority (70%–90%) of disease-associated mutations in HCM are identified in three genes encoding elements of the cardiac sarcomere. These include myosin binding protein C (*MYBPC3*), β-myosin heavy chain (*MYH7*) and troponin T (*TNNT2*), all of which are associated with classic, autosomal dominant inheritance [91–93, 95]. However, HCM has important phenocopies that may not be immediately obvious clinically, or following routine cardiac pathology evaluation (Table 2). These genetic syndromes, including Fabry disease (*GLA*) and Danon disease (*LAMP2*) among others, can be associated with multi-systemic medical issues resulting in risks for additional, extra-cardiac conditions and different management recommendations for at-risk relatives [94]. Some of these conditions also follow other modes of inheritance and in clarifying the underlying aetiology, will allow for targeted family screening. In the case of X-linked genetic disorders, it may be possible to eliminate the necessity for clinical surveillance for some relatives such as sons of affected males, while ensuring appropriate multi-system surveillance for those truly at risk [93, 94, 96]. Identification of a disease-causing variant can also be helpful in targeting appropriate individuals for clinical surveillance. As per international guidelines, a (definite or suspected) clinical diagnosis of HCM prompts family screening including serial echocardiograms and ECGs for all first degree relatives of an affected individual [90, 97]. Clinical surveillance is essentially life-long, with repeated evaluations recommended to occur every 3–5 years starting at age 10 [90, 98, 99]. If, through molecular autopsy, a causative gene mutation is identified, subsequent cascade genetic testing can be offered to all at-risk family members, including more distant relatives who may be at risk but who would not typically be offered clinical screening. Those found to carry the high-risk mutation can be appropriately screened and those who test negative may not require such intense clinical follow-up [93, 94, 96, 98]. While the hereditary nature of HCM is well-recognized in the medical community, families may sometimes find this difficult to reconcile with their own lived experience, particularly in the absence of positive family history for HCM or sudden death. Cann et al. [100] recently demonstrated that family members were more likely to undergo clinical evaluations if a molecular diagnosis was made at the time of autopsy, highlighting an important value to postmortem genetic testing in HCM and other inherited cardiomyopathies. While a postmortem diagnosis of HCM can, on its own, prompt the appropriate clinical surveillance recommendations for family members, there is value in molecular autopsy. Given that the identification of a causative gene mutation provides accurate diagnosis for the family, as well as access to predictive genetic testing for at-risk relatives, postmortem genetic testing is a reasonable consideration when LVH is identified as the cause of sudden death.

**Table 2.** Overview of genes associated with hypertrophic cardiomyopathy/left ventricular hypertrophy.^a^

Inheritance	Genes	Detection
Non-syndromic, AD	*MYBPC3*^b^, *MYH7*^b^, *TNNT2*, *TNNI3*, *ABCC9*, *ACTC1*, *ACTN2*, *CSRP3*, *MYL2*, *MYL3*, *MYOZ2*, *NEXN*, *TNNC1*, *TPM1*, *PRKAG2*, *CAV3*, *JPH2*, *PLN*, *CALR3*, *LDB3*, *TCAP*, *VCL*, *ANKRD1*, *MYPN*	25%–60% [93]
Syndromic, AD	*TTR*, *RAF1+*, *PTPN11+*	1%–7% [101, 102]
Syndromic, XL	*GLA*, *LAMP2*	1%–3% [103, 104]

^a^This table represents genes that have been reported in association with various clinical phenotypes, however, given the dynamic nature of disease-gene associations it is not an exhaustive list. Genes that are included on individual genetic testing panels vary between laboratories. AD: autosomal dominant; XL: X-linked.

^b^Accounts for >5% of cases.
